# Should all excised surgical specimens be examined microscopically? A case report

**DOI:** 10.1186/1757-1626-3-40

**Published:** 2010-01-28

**Authors:** Thomas M Raymond, Sam Ibrahim, Pradeep S Basnyat

**Affiliations:** 1 Surgical Department, William Harvey Hospital, Kennington Road, Ashford, Kent, UK

## Abstract

**Background:**

Microscopic examination of all surgical specimens is controversial.

**Case Presentation:**

We report two cases where examination has revealed unexpected results that have changed patient management and treatment.

**Conclusions:**

Histopathological examination of all tissue should be considered. The benefits and disadvantages of routine examination of tissue are discussed.

## Introduction

Most surgeons routinely send surgical specimens for microscopic examination, however there is reluctance among some surgeons to send specimens, especially from minor operations such as cysts and lipomas. This may reduce pathology workload and costs. However the histological diagnosis will remain uncertain and the surgeon will lose an element of feedback and quality control. We report two cases to illustrate that all surgical specimens should be considered for histological examination.

## Case 1

An eighty one-year-old lady presented to the emergency department with a right groin swelling for more than two years which had become acutely painful. Clinical diagnosis of a femoral hernia was made. At operation, the sac contained a knuckle of non-viable omentum which was otherwise unremarkable. This was excised and was sent for histopathological examination. The histology revealed a metastatic papillary adenocarcinoma, moderate to poorly differentiated, with a probable ovarian primary. Postoperative CA125 was markedly elevated at 853.2 KU/L (normal value less than 30 KU/L). Subsequent ultrasound scan of her pelvis showed an 84 × 47 × 80 mm solid mass of probable ovarian origin in the pouch of Douglas. The patient recovered well from her surgery and was subsequently referred to the gynaecologists for further management.

## Case 2

A forty-year-old female had a routine excision of a sebaceous cyst from the anterior chest wall, as day case, under local anaesthesia. The specimen was sent for microscopic evaluation and was reported as a sebaceous adenoma. This rare skin neoplasm when combined with a family history of colorectal cancer suggests Muir-Torre syndrome (This is a rare autosomal dominant genodermatosis recognised as a subtype of LynchType II hereditary nonpolyposis colorectal cancer), which increases the risk of developing bowel, uterine and other visceral malignancies [1].

As per recommendation by a geneticist she is under long-term follow-up by surgeons and gynaecologists [2]. As this is an autosomal dominant condition it has implications with regard to screening of her relatives [1,2].

## Discussion

A number of groups have recommended that routine surgical specimens should be discarded and not sent for histological microscopic examination unless macroscopically abnormal. These include paediatric hernia sacs (no unexpected findings in 1494 and 371 specimens in two studies [3,4]), gallbladders (all adenomas and carcinomas macroscopically abnormal and suspected at the time of surgery in 1523 gallbladders [5]), and haemorrhoids (3 found in 311 specimens and all suspected at operation [5]).

There is debate in the literature regarding other tissues. Kassan et al found 1 in 1020 adult hernia sacs contained an unexpected abnormality and suggested this was too rare a finding to advise examination of specimens routinely [6]. Connelly et al [7] suggest sending all hernia sacs for histopathological examination to exclude malignancy to exclude these rare findings.

These two reported cases highlight the importance of sending excised tissue for microscopic examination despite the rarity of the findings. It is advisable to maintain a high index of suspicion (previous intraperitoneal malignancy and elderly patients who develop incarceration of a chronic hernia) [8], examine the specimen for macroscopic abnormality and send tissue for microscopic examination. This will confirm the diagnosis and avoid missing unexpected malignancy [9] and the medico legal consequences.

## Competing interests

The authors declare that they have no competing interests.

## Consent

The patients in this report are untraceable, there is no reason to believe that the patients would object and they are completely anonymous. The cases provide a background for discussion of the topic only.

## Authors' contributions

TR - Wrote paper

SI - Collected patient information and performed literature search

PB - Original idea reviewed and altered paper.

All authors read and approved the final manuscript
